# Multi-country evidence on societal factors to include in energy transition modelling

**DOI:** 10.1038/s41560-025-01719-7

**Published:** 2025-02-21

**Authors:** Vivien Fisch-Romito, Marc Jaxa-Rozen, Xin Wen, Evelina Trutnevyte

**Affiliations:** 1 Renewable Energy Systems Group, Section of Earth and Environmental Sciences, University of Geneva, Geneva, Switzerland; 2 Present Address: Institute of Geography and Sustainability, Faculty of Geosciences and Environment, University of Lausanne, Lausanne, Switzerland; 3 Present Address: Joint Research Centre, European Commission, Seville, Spain

**Keywords:** Energy supply and demand, Socioeconomic scenarios, Climate-change mitigation

## Abstract

Integrated assessment and energy system models are challenged to account for societal transformation dynamics, but empirical evidence is lacking on which factors to incorporate, how and to what extent this would improve the relevance of modelled pathways. Here we include six societal factors related to infrastructure dynamics, actors and decision-making, and social and institutional context into an open-source simulation model of the national power system transition. We apply this model in 31 European countries and, using hindcasting (1990–2019), quantify which societal factors improved the modelled pathways. We find that, if well-chosen and in most cases, incorporating societal factors can improve the hindcasting performance by up to 27% for modelled installed capacity of individual technologies. Public acceptance, investment risks and infrastructure lock*-*in contribute the most to model performance improvement. Our study paves the way to a systematic and objective selection of societal factors to be included in energy transition modelling.

## Abstract (web summary)

Mitigation scenarios are required to account for societal factors. Fisch-Romito et al. integrate factors related to infrastructure, actors’ decision-making and socio-institutional context into a national energy model, and, using hindcasting, show how this can improve the modelled pathways.

## Main

Integrated assessment models of climate change and energy system models are widely used to quantify low-carbon pathways at global and national scales. For the power sector, which needs to decarbonize earlier than other sectors^1^, the models represent electricity supply, end-use technologies, service demands and their interlinked dynamics to assess the sector’s transformation to enable broader carbon neutrality. In practice, this transformation depends on the co-evolution of technical, social, economic, institutional or political factors, which may induce lock-ins or tipping point dynamics and change feasibility space for the emergence of new technologies^2–4^. Modellers are now increasingly requested to account for societal transformation dynamics in their modelled pathways to inform policymakers about intervention designs, their feasibility and impact^5–9^. Integrating the societal elements in models is crucial to avoid biased policy recommendations that are based on technical or economic criteria only^9^ and to better assess the plausibility of achieving future climate objectives^10^.

Social scientific knowledge on energy and climate mitigation has so far evolved in parallel to modelling and analysis of pathways^11^ with rare links done either ex ante with informed storylines^12^ or ex post by evaluating pathways’ outputs on different societal indicators^13^. The few articles that have attempted to represent societal factors of power sector transitions in technology-rich models have focused on one factor at a time, such as cost of capital^14^, actors’ behaviour^15,16^ or social acceptance^17^. As a result, critiques of models have emerged in the meantime about their poor ability to produce solutions-oriented outcomes or to inform about feasibility^11,18,19^. On the one hand, optimization models, which are based on an intertemporal cost minimization with perfect foresight approach, have shortcomings to capture any societal factors and especially processes that influence investment decision-making, such as mutual influence between actors and path dependency^20,21^. On the other hand, analyses that endogenize multiple societal factors into simulation rather than optimization models could contribute to responding to these critiques, but such models, although common in policy analysis^22^, are at the early stage of development^10,23,24^.

Most of all, it is unclear whether some societal factors should be given priority over others for inclusion in models and how these factors should be endogenized (see, for instance, recent methodology description for the integration of capital costs^25^). Critiques have pointed out the limited performance of models in capturing real-word transitions^26,27^, but it is unclear to what extent the incorporation of societal factors would improve it or not. This relates to model evaluation, which is an emerging topic in the energy and integrated assessment modelling community to improve robustness and usefulness of models for policymakers^28^. Hindcasting—assessing model ability to reproduce past dynamics—is one relevant evaluation method to assess the appropriateness and credibility of a model structure^28^. The few hindcasting exercises that have been done so far^27,29–31^ highlight that the difficulty to capture investment dynamics in power sector models is a general issue, regardless of the model type. To the best of our knowledge, no hindcasting exercises have yet considered the integration of societal factors.

Here we provide empirical evidence using an open-source techno-economic simulation model of the power system transition with a similar structure to some integrated assessment and energy system simulation models (for example, BLUE^24^, GCAM^32^, IMACLIM^33^, IMAGE^34^ or POLES^35^) where we incorporate, one by one and in combination, six societal factors related to infrastructure dynamics, actors and decision-making, and social and institutional context (Table 1). We set up this model for 31 European countries (EU27, Switzerland, Iceland, Norway and the United Kingdom) to allow generalizable insights and conduct a hindcasting evaluation of power capacity expansion, renewable generation and CO_2_ emissions from 1990 to 2019. This approach allows us to conclude on which societal factors and how should be ideally incorporated in future modelling.

**Table 1:** Societal factors included in the model one by one and in combination

Aspects	Societal factors included	Approach how this factor is represented in the model
Infrastructure dynamics	Lock-in (L)	Power capacity lifetime is extended by up to 10 (20; 30) years after the end of technical lifetime if this capacity is economically profitable to keep using in the future at the same utilization rate.
	Fast transition (T)	Existing capacity is retired before the end of the technical lifetime if it is economically profitable to build and use alternative technologies at the same utilization rate. This capacity that is retired earlier for each technology is limited annually to 2% (5%; 10%) of the total installed capacity.
Actors and decision-making	Actor heterogeneity (A)	Lower cost sensitivity is assumed for investment choices^24^. Between two available technologies, a cost saving of 20% results in a 75% (60%; 90%) market share instead of 100%.
	Investment risks (I)	Different weighted average costs of capital are used per country and technology^70^.
Social and institutional context	Public acceptance (P)	Incumbent technologies and wind power capacity cannot be built when more than 60% (50%; 70%) of the population has a negative perception of them^62^. For wind power, the local acceptance is assumed to be 35% lower than overall public acceptance to represent disamenity costs^17,64^. Wind and solar power investment costs are reduced by 20% if more than 80% of the population has a positive perception of these technologies.
	Governance (G)	The combination of electricity market liberalization and state-owned utilities facilitates the emergence of renewable technologies^39,40^. When entry barriers are low and public ownership is high, a 20% (10%; 30%) decrease in renewable investment costs is assumed. Conversely, when entry barriers are high and public ownership is low, a 20% (10%; 30%) increase in investment costs is assumed.

Methods provides more details on model implementation of these factors. Numbers in brackets refer to alternative parameter values tested for sensitivity analysis.

## Societal factors improve the model’s hindcasting performance

**Fig. 1:**
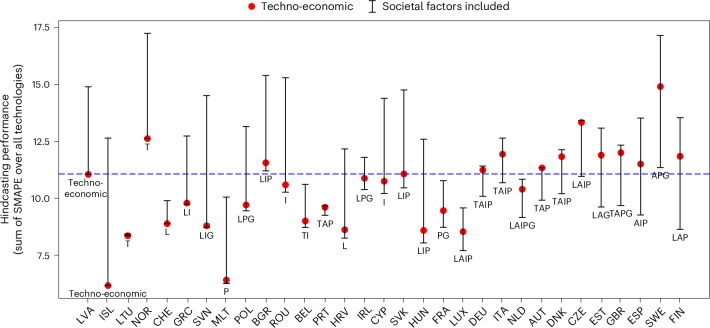
Comparison of the hindcasting performance of the techno-economic model and all model versions with societal factors included for 1990–2019 in terms of installed capacity. Hindcasting performance corresponds to the values of SMAPE over the years summed over all technologies. The lower (higher) is the value, the better (worse) is the hindcasting performance. Red dots refer to the techno-economic model version without societal factors incorporated. Black bars refer to the range of model version with societal factors included one by one and as combinations. With each societal factor represented or not, the range of hindcasting performance for each country is based on 7 to 64 hindcasting simulations depending on data availability (see Supplementary Fig. 1 for details about the combinations of factors considered). Data are presented as the range between the minimum and maximum values. Countries are ordered by gain in hindcasting performance induced by the inclusion of societal factors compared with the techno-economic model. Country names are represented with three-letter codes defined by the International Organization for Standardization (ISO) 3166-1 Alpha-3 country codes. The blue line represents the average hindcasting performance of all combination cases of countries and societal factors. For each country, the combination of societal factors leading to the best hindcasting performance is highlighted. Each letter refers to one societal factor incorporated: L, lock-in; T, fast transition; A, actors heterogeneity; I, investment risk; P, public acceptance; G, governance.

We find that incorporating societal factors in the model one by one or as combinations mostly improves the hindcasting performance in terms of installed capacity dynamics for most of the 31 European countries studied (Fig. 1). Only in the cases of Iceland and Latvia, the model version with the best hindcasting performance (that is, lowest error) is the techno-economic one without societal factors incorporated. For the rest, the model version with the best hindcasting performance includes at least one societal factor. We obtain high heterogeneity between countries in terms of hindcasting performance gain compared with the techno-economic model. Minor gain in hindcasting performance is achieved for Greece, Lithuania, Norway, Slovenia and Switzerland (less than 1% of symmetric mean absolute percentage error (SMAPE)) and low gain (less than 5%) for another eight countries (Belgium, Bulgaria, Croatia, Ireland, Malta, Poland, Portugal and Romania). Countries with minor gains are characterized by low cumulative installed capacities in 1990, below 10 GW, and a limited diversity of technologies, or by a high proportion of hydroelectric power plants, which induces inertia in the evolution of the system owing to long lifetimes. Values for Austria, Cyprus, Denmark, France, Germany, Hungary, Italy, Luxembourg, Netherlands and Slovakia lie in between 5% and 15%. We obtain the highest hindcasting performance gain of more than 15% of SMAPE for six countries (Czech Republic, Estonia, Finland, Spain, Sweden and the United Kingdom). This is mainly because, contrary to the techno-economic model, the inclusion of some societal factors in the model allows to better capture the increase in installed capacities of decentralized renewable technologies (for example, biomass, offshore and onshore wind and solar photovoltaic) observed in these countries from 2010.

Even if the inclusion of one or several societal factors can improve the hindcasting performance for most of the countries, there are many cases when including some specific factors or their combinations, the hindcasting performance gets worse than the performance of the techno-economic model (Fig. 1). Societal factors hence need to be included in an evidence-based way to avoid worsening the model’s performance and hindcasting is a relevant approach to do so. We also observe a large spread in terms of absolute hindcasting performance (Fig. 1). On the one hand, for Belgium, France, Lithuania, Luxembourg, Malta, Netherlands, Portugal and Switzerland, the model version with the worst hindcasting performance has a better performance than the average of the whole simulation set, all countries considered. Except for France, Luxembourg and Netherlands, low performance gains are obtained for these countries as well, pointing to a tiebreak between additional modelling efforts and actual gain. On the other hand, for Bulgaria, Norway and Sweden, the model version with the best hindcasting performance gives more errors than the average value of the whole hindcasting simulations set. For this second group of countries, this suggests a potential for improving the model structure and the way that societal factors are incorporated to improve the model performance to capture capacity dynamics.

In relation to this statement, we also perform a sensitivity analysis on the key assumptions used to represent the actors heterogeneity, lock-in, fast transition, public acceptance and governance societal factors (Table 1). For lock-in, fast transition, public acceptance and governance societal factors (Table 1), we find that the direction of accuracy variation in relation to the techno-economic model remains the same except with public acceptance for Estonia, Norway and Portugal and with governance for Norway (Supplementary Figs. 3–6). For Estonia, integrating public acceptance, with the assumption of a 40% population threshold supporting a technology below which the technology in question can no longer be built, improves the hindcasting performance. The hindcasting performance is reduced when a 30% population threshold is used because investments in nuclear start earlier in the model, whereas there is no nuclear capacity in historical observations. For Portugal, the hindcasting performance decreases in the 50% population threshold case but increases in the 40% population threshold case because the latter allows more investments in onshore wind capacities, which is more in line with historical observations. For Norway, integrating public acceptance with a 40% population threshold decreases the hindcasting performance but increases it with a 50% population threshold because it prevents investments in nuclear capacity such as in historical observations. The hindcasting performance decreases for governance when wind investment costs are reduced by 20%, but increases when reduced by 10% because in the first case, there are investments in both offshore and onshore wind whereas there is no installed offshore wind capacity in historical observations. For actors heterogeneity, the accuracy is more sensitive to the parameter we vary, the cost sensitivity of investments, compared with the parameters varied for other societal factors (Supplementary Fig. 7) because cost sensitivity drives the shares of new investments for all technologies. Also, the direction of accuracy variation compared with the techno-economic model is modified for seven countries according to the cost sensitivity value chosen. This calls for carefully calibrating in future scenarios this parameter, widely used in some established models^32,34^, to obtain consistent capacity dynamics.

## Societal factor’s contribution differs by country and factor

The selection of societal factors leading to the best hindcasting performance differs between countries (Fig. 1). First, there is a difference in terms of the number of societal factors incorporated, ranging from one for Croatia, Cyprus, Lithuania, Malta, Norway, Romania and Switzerland to five for Netherlands. Except the latter, there is no country where the integration of more than four factors leads to an additional gain in performance. This suggests that increasing the complexity of the model does not necessarily increase its accuracy, supporting previous findings on complexity of technology representation in models^36^. Second, the best combinations differ in terms of societal factors incorporated. Each societal factor is present for at least eight countries in the model version giving the best hindcasting performance, which suggests that all societal factors analysed are potentially relevant to incorporate in integrated assessment and energy system models. Public acceptance, investment risks and lock-in are the societal factors most present in the model versions giving the lowest errors, with respectively 18, 15 and 14 occurrences over 31 countries (Fig. 1).

**Fig. 2:**
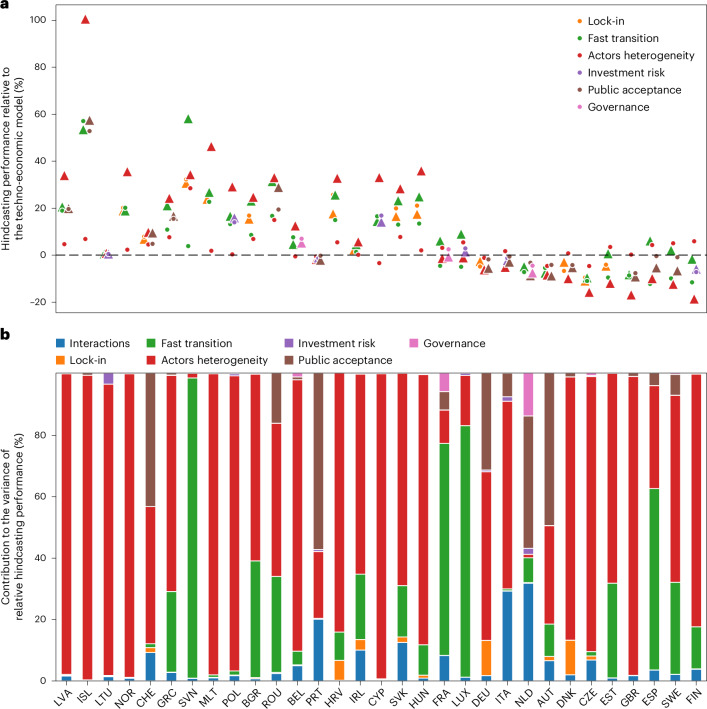
Influence of societal factors on model hindcasting performance for installed capacity. **a**, The direction and size of the effect of each societal factor. **b**, The analysis of variance on model hindcasting performance. Hindcasting performance corresponds to the SMAPE over the years summed over all technologies. In **a**, the triangle represents the average relative model performance across the hindcasting simulation subset with the considered societal factor included. The point represents the average relative model performance across the hindcasting simulation subset without the considered societal factor included. Only the three most influencing factors are represented for readability. All factors are represented in Supplementary Fig. 2.

Model hindcasting performance is more sensitive to the incorporation of some societal factors in the model than others, both positively and negatively. Actors heterogeneity has the highest influence in most countries (Fig. 2b), which is expected since this factor accounts for cost sensitivity, which, in turn, drives the shares of new investments for all technologies. The second most influencing factor overall is the fast transition factor, which represents more than 20% of the variance for ten countries (Fig. 2b) but tends to decrease the hindcasting performance for them (Fig. 2a). Public acceptance is the third most influential factor representing more than 10% of the hindcasting performance variance in six countries (Austria, Germany, Netherlands, Portugal, Romania and Switzerland), but with a negative effect on model hindcasting performance for Switzerland and Romania (Fig. 2a,b). This highlights the importance to be cautious when introducing or omitting these three factors because they are not always beneficial.

Interactions of certain societal factors also have a high influence on hindcasting performance for Italy and Netherlands for which they represent more than 25% of the variance (Fig. 2b). In the case of Italy, this is mainly due to a synergistic effect of fast transition and actors heterogeneity that gives an overall hindcasting gain greater than the sum of the effects of each factor individually (Supplementary Fig. 1). Conversely, for the Netherlands, we obtain antagonistic effect with the combination of public acceptance and governance and with actors heterogeneity and governance, which together give lower performance than the sum of the effects of each factor individually (Supplementary Fig. 1). Remarkably, the integration of any societal factor, one by one or in combination, has limited influence on the model hindcasting performance for Lithuania and Portugal (Fig. 2a). Lithuania is characterized by an oversized supply system compared with its electricity demand with low technology diversity, creating a locked dynamics in all modelled pathways. For Portugal, the much lower costs for coal and wind power compared with other technologies drive the capacity dynamics in the model.

## Hindcasting performance trade-off between model outputs

**Fig. 3:**
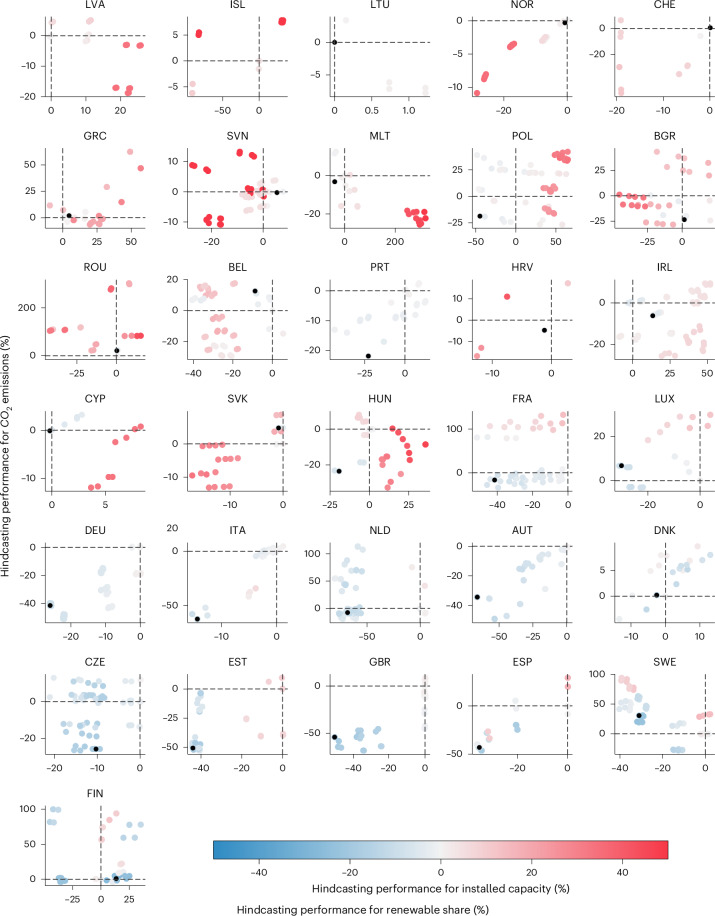
Comparison of the hindcasting performance (relative to the techno-economic model) for installed capacity, share of renewable electricity production and CO_2_ emissions for each country studied. Each dot refers to a model version with one combination of societal factors incorporated. The black dot represents the model version with the best hindcasting performance for installed capacity.

For most countries, the combination of societal factors giving the lowest deviation from historical data for capacity dynamics is not the same as giving the lowest deviation for CO_2_ emissions and the share of renewable electricity production (Fig. 3). We hence obtain a trade-off in hindcasting performance between model outputs but to a different extent between countries. For a first group of 17 countries composed of Austria, Bulgaria, Croatia, Cyprus, Czech Republic, Estonia, France, Germany, Hungary, Italy, Malta, Netherlands, Norway, Poland, Portugal, Spain and the United Kingdom, the model version giving the best hindcasting performance for installed capacity also improves the model performance for the 2 other outputs compared with the techno-economic model. For the second group of 11 countries composed of Belgium, Denmark, Finland, Greece, Ireland, Luxembourg, Romania, Slovakia, Slovenia, Sweden and Switzerland, the model version giving the best hindcasting performance for installed capacity gives more errors than the techno-economic model for at least 1 of the other outputs. This suggests the need to use a combination of societal factors, like a country-specific pareto-optimal set of factors, that altogether would allow to improve the hindcasting performance for the three metrics, and other relevant model metrics too. For Belgium, Denmark, Finland, Luxembourg and Sweden, there are combinations of societal factors that are relevant to improve the performance for the three model outputs. Conversely, this is not the case for Greece, Ireland, Romania, Slovakia, Slovenia and Switzerland suggesting the need for these countries to better represent the societal factors or to further modify the original structure of the model.

## Discussion

This study provides systematic evidence to further incorporate societal factors into integrated assessment models and energy system models to construct low-carbon transition pathways. By doing a multi-country hindcasting exercise with a simulation model of the power sector with a similar structure to integrated assessment and energy system simulation models (for example, BLUE^24^, GCAM^32^, IMACLIM^33^, IMAGE^34^ or POLES^35^), we draw implications for the future development of these models and related scenarios. We find that incorporating societal factors related to infrastructure dynamics, actors and decision-making, and social and institutional context has the potential to improve the model performance to capture the real-world dynamics of installed capacities of individual technologies in the power sector. This finding is valid for most of the European countries studied, but the combination of societal factors incorporated to increase the hindcasting performance differs between countries. Actors heterogeneity influences the model outputs the most, but public acceptance, investment risks and lock-in of infrastructure are most likely to be associated with a best hindcasting performance. Modellers should hence focus their future research on these four societal factors for integration into models.

We show that the gain in model performance induced by the integration of societal factors to capture power system dynamics depends strongly on the national energy context. Low performance gains are obtained for countries with small cumulative installed capacities and with high shares of hydroelectric production in their electricity mix. Conversely, high performance gains are obtained for countries that tend to have high cumulative installed capacities and that have experienced in the past decade rapid growth in decentralized renewable technologies. During the period 1990–2019, various technologies were also at different stages of maturity and were therefore influenced by societal factors in different ways. As the uptake of new electricity technologies is often erratic^37^ and highly dependent on context-specific factors^38^, the integration of societal factors could help to better capture this formative phase. We also highlight that a performant model structure for installed capacities is not necessarily relevant to capture other output dynamics, such as renewable generation or CO_2_ emissions, with a potential accuracy trade-off. Going further, these elements suggest that there is no one-size-fits-all approach and that models that represent national energy transitions should be more country specific in building their structure and should be evaluated on different outputs to choose one or several adequate model structures. Hindcasting exercises are key here to provide evidence for model construction. Having said that, our results also reveal that this high variation in country contexts poses challenges to drawing generalizable explanations for model hindcasting performance.

Our hindcasting study was limited to six societal factors frequently mentioned in the literature as influencing investments and installed power capacity dynamics. Our results are naturally sensitive to the chosen indicators to proxy these factors and in the future one could test alternative model implementations^9^. For instance, we chose two indicators to proxy governance, namely entry barriers for access to competitive electricity markets and ownership by state entities since there is empirical evidence to relate these factors with investment patterns in electricity generation^39,40^. But one could also test other indicators such as state’s functioning capacity^41^ or functioning-of-government index^42^, used in recent research on coal capacity dynamics. In the future, testing the impact of other societal factors or other model implementations of the same societal factors could also be relevant, not only for countries where societal factors decreased the model performance but also to improve the model performance simultaneously on multiple outputs for countries where we obtain a high-performance trade-off. Second, including the factors influencing electricity demand would be interesting to better capture the co-evolution of electricity demand and supply that has led to high overcapacity for some European countries^43^. Capturing this co-evolution is all the more important at a time when energy security and demand reduction are back in the spotlight because of the conflict in Ukraine. Finally, future work should include societal factors and test the hindcasting performance of the whole energy system models and eventually of integrated assessment models of climate change. Beyond simulation models, endogenization of societal factors into optimization models^44^ could also be tested to potentially extend these insights to another family of models.

Energy modellers are always torn between proposing the most realistic model structure possible for policy relevance and at the same time making simplifications to be able to interpret and communicate the results. This study confirms that increasing model complexity (that is, incorporating more societal factors) is not equal to increasing model accuracy and performance^36^. The hindcasting methodology could be replicated during any model building phase^44^ to obtain a parsimonious model using as few explanatory variables as possible while still ensuring that the model is ‘good enough’ for its specific purpose^45,46^. Moreover, this study highlights that the modellers’ decisions to incorporate or not to incorporate societal factors are impactful for results. The hindcasting methodology allows to disentangle the societal factors that most influence the model performance by themselves or in interaction with other factors. The exercise done here hence paves the way to reduce subjectivity of modeller’s decisions^47^ and to be transparent on the structural uncertainties of the model^28^. We also provide tools and datasets in this paper and elsewhere^29,48^ to enable informed choice.

This study supports that further efforts should be devoted to two aspects on model building methodology. First, introducing societal factors in integrated assessment and energy system models can substantially improve their performance, which calls for a stronger cooperation of this community with social sciences to have representations of societal factors more empirically based. This can be challenging in practice because it implies bringing together researchers from different epistemic communities and methodological approaches. For instance, some model structures coming from engineering may not be easily adapted in their current state for including societal factors and, when relevant, may raise issues of computational demand and results tractability. There is also a potential issue of data availability in some geographical areas, notably in the Global South where political and social aspects of energy transition have been understudied so far^49^.

Second, integrated assessment and energy system models have been mostly ‘diagnosed’ so far through prospective intercomparison exercises to identify their main assumptions, characteristics and behaviour^50,51^. To go further, we argue that this should be completed by common hindcasting exercises, as done in this Article, to highlight their (in)ability to capture sectoral and national dynamics and adapt the model structure. However, a research project with large-scale hindcasting experiments would not necessarily produce policy-relevant outputs, which may be challenging for raising funding. Reproducing past trends in hindcasting is not the guarantee of a better model performance because energy–economy–social systems do not exhibit structural constancy over time^46,52^. However, a model that is good enough to capture past trends can produce reference case scenarios within a similar context to better define the feasibility space^4^. This is crucial to give elements for discussing not only the techno-economic but also socio-political feasibility of achieving climate targets^53,54^ and to highlight how current socio-technical dynamics potentially hinders climate ambition in the short term and what policies are needed^55^.

## Methods

### Summary

In our paper, we set up an open-source model of the power system transition (‘The STONES model’ section) and followed a two-step modelling approach. In the first step, we developed a set of hindcasting simulations of national power generation and capacity expansion in 31 European countries (EU27, Switzerland, Iceland, Norway and the United Kingdom) from 1990 to 2019. Each simulation corresponds to a model version where one or a combination of several societal factors from Table 1 are included (‘Representation of societal transformation factors’ section). In the second step, from each scenario, we extracted the trends in terms of dynamics of installed capacity, generation and CO_2_ emissions and compared them to historical pathways to evaluate the hindcasting performance. The ‘Hindcasting performance’ section describes the metrics used to evaluate hindcasting performance.

### The STONES model

For the purpose of this study, we built an open-source simulation model of the power system, called Socio-Technical Outlook of National Energy System (STONES). STONES is a bottom-up, technology-rich, simulation model with a recursive dynamic of 1 year time step. The model can be considered as a socio-technical energy transition model since it represents techno-economic detail, actor heterogeneity and transition pathway dynamics^56^. It includes 14 different electricity production and storage technologies. The model takes as inputs national energy systems data such as costs, resource potentials and flexibility constraints for each technology. The outputs are the installed capacities of individual technologies and electricity production for every year for all technologies. The STONES model is composed of three distinct modules used consecutively at each time step: the electricity demand module, the dispatch module and the capacity expansion module, all described in detail in Supplementary Section 1.

The electricity demand module builds load duration curves for the current year and the expected ones in the future. It allows to obtain hourly electricity demand values for the observed year to feed the dispatch module, and for 5 years ahead to feed the capacity expansion module. The model uses as inputs the hourly electricity demand profiles and the hourly load factors of non-dispatchable renewable technologies for the current year. The annual time series are aggregated using clustering into six representative days to reduce calculation time because of computational constraints.

The dispatch module determines the system-wide cost-optimal dispatch by minimizing the sum of variable costs for all technologies while satisfying hourly electricity demand constraints over the six representative days obtained from the electricity demand module. This optimization is done satisfying different constraints on electricity production, adequacy and transmission capacities.

The capacity dynamic module determines the evolution of installed power capacity to satisfy the projected electricity demand 5 years later. As main inputs, the model uses technology costs, age of installed capacity and expected load duration curves obtained from the electricity demand module. First, the module estimates the total capacity needed to satisfy the expected peak demand 5 years later based on the expected capacity retirement over the following years. Then, since only a part of the installed capacity generates power at full load all year, the module divides the load duration curves into load investment segments (that is, peak load, intermediate load and base load) to make the link with dispatch decisions, following previous approaches^32,34,35^. The total needed capacity is then split between technologies using a multinomial logit equation, a common approach in energy simulation models^8,34^. To do so, for each investment segment, the technologies are compared based on their annualized levelized costs. Finally, the capital stock evolves at each time step and for each technology, based on new investments made and retirement of the existing capacity according to the lifespan.

### Representation of societal transformation factors

In the STONES model, we integrated six societal transformation factors related to three influencing aspects of energy transition, namely infrastructure dynamics, actors and decision-making, and social and institutional context (Table 1). Infrastructure dynamics influence the transition speed of existing capital towards new technologies. On the one hand, power infrastructure has long lifespan and is characterized by lumpiness and a path-dependent evolution^57,58^. This lock-in effect factor incentivizes the usage of existing capacity for a longer time. On the other hand, the market environment can incentivize earlier retirement of capacities, inducing a more flexible infrastructure dynamics and allowing a fast transition towards alternative power technologies. We incorporate these two factors in the model for coal, gas and nuclear technologies since they have represented an important share of the electricity mix in the past decades and because capacity age data were available. We represent lock-in in the model by allowing a lifetime extension of 10 years. We also perform a sensitivity analysis by testing the cases with a lifetime extension of 20 and 30 years. To do so, at the expected lifespan end of incumbent capacity, the model compares the costs of continuing to use the incumbent capacity (that is, annual operational costs) with the costs of building and using new capacity of alternative technologies (that is, annual levelized costs). To represent fast transition, a similar comparison is done in each year in the model. In main model runs, the earlier retired capacity is limited annually for each technology to 2% of the total installed capacity. We also perform a sensitivity analysis by testing the cases with a 5% and a 10% annual limit. The cost comparisons are done for both societal factors, assuming that potential alternative technologies would produce electricity in a similar pattern to avoid, for instance, inconsistent replacement of a base load technology by a peak technology. We use a multinomial logit equation for this comparison to allow partial replacement and extension of the capacities.

When incorporating the aspects of actors and decision-making, we modified the perceived technology costs and hence influenced the direction of power capacity transition. First, there are various actors involved in the process of capacity investment decision-making, and some of them allocate a lower weight to technology cost for their decision. We represent actors heterogeneity in the model by decreasing the cost sensitivity of investment choices in the multinomial logit equation^16^. In the main runs, we assume that between two available technologies, a cost saving of 20% results in a 75% market share instead of 100%. We also perform a sensitivity analysis by testing the cases where a cost saving of 20% results in a 60% and 90% market. Second, investment risks quantified by the cost of capital appear as an important driver of investment choices, especially for technologies with high upfront costs, such as renewable generation^59^, and it evolves over time and differs between countries^60^. Following the methodology of Steffen^14^, we represent risk in the model by replacing the discount rate with the weighted average cost of capital in the levelized cost calculation differentiated by country, technology and observed year.

The social and institutional contexts create nonlinear change in energy transition by either continuing past trends of investments or, with changes beyond a certain threshold, speeding up the uptake of new technologies. First of all, public acceptance influences the emergence of renewable generation in the energy landscape. Wind energy diffusion has recently slowed down in some countries owing to public acceptance issues^61^. Negative public opinion about incumbent technologies can lead to a technology phase-out such as nuclear power in Germany during the 2010s, creating a window of opportunity for the emergence of renewable technologies. To represent this stylized fact, we assumed that a technology cannot be built in the model for a given year when more than 60% of the population has a negative perception of it, following Cotterman et al.^62^. We also perform a sensitivity analysis by testing the cases with 40% and 70% population thresholds. For wind power, local acceptance of wind projects is a more important factor than overall public opinion because of potential disamenity costs^17,63^. Based on data from previous studies^17,64^, we assumed that local acceptance is 35% lower than overall public acceptance for wind power and then used this figure to apply the same approach as for other technologies to represent power technologies rejection. Conversely, widespread positive opinion about renewable generation technologies over the population creates pressure on governments for the adoption of support policies^65^. To represent the variation in perception of adoption costs, we assumed that wind and solar investment costs are reduced by 20% if more than 80% of the population has a positive perception of these technologies. Public opinion data on electricity generation technologies come from Eurobarometer surveys carried out by the European commission in 1991, 1993, 1997, 2002, 2006, 2007, 2010 and 2012, the European Social Survey Round 8 carried out in 2016 and the Global Snap Poll carried out by WIN-Gallup International in 2011 (see detailed sources by technologies in Supplementary Table 1).

Second, governance of national power infrastructure and markets tends to modify the investment patterns. Energy liberalization has increased public support for renewable energy in Organisation for Economic Co-operation and Development (OECD) countries, mainly because of reduction in entry barriers^40^. State-owned utilities show higher tendency to invest in renewable generation^39^. We represent these trends in the model by modifying as well the investment cost for solar and wind technologies in relation to two governance indicators obtained from the OECD Product Market Regulation database^66^: legal or administrative barriers for third-party access to competitive electricity markets (entry barriers) and ownership or direct control of transmission and generation enterprises by state entities (public ownership). Indicator values range from 0 to 6. When entry barriers are low (≤2) and public ownership is high (≥4), we assumed a 20% decrease of investment costs. Conversely, when entry barriers are high (≥4) and public ownership is low (≤2), we assumed a 20% increase of investment costs. We also perform a sensitivity analysis with a 10% and a 30% decrease of investment costs.

### Historical data

Most of the historical data about costs, generation and capacity dynamics come from the same data package^48^. It covers the period of 1990–2019 for the 31 European countries used in this Article. Original data sources were identified using a literature review focused on open-access sources, energy company statistics and national agency statistics. These original data sources were then harmonized to enable their use as input parameters for power system modelling. This approach produced four types of processed data file for each country: a country file documenting nationally aggregated variables, such as annual time series of electricity demand, transmission and distribution losses, and peak load; technology files describing techno-economic variables for each major generation technology in the country’s electricity mix, such as annual time series of investment costs, installed capacities and actual generation; resource files describing CO_2_ emissions intensities and annual time series of costs for each generation fuel or input resource; and load profiles describing 24-hour national load curves for each available year. The data package includes an annotated list of all used original data sources, describes harmonization and processing steps for each variable, and provides the final processed data files in comma-separated format. Other input data and sources used to represent the societal factors are described in the main text (Table 1), Methods and Supplementary Table 1.

### Hindcasting performance

For each country, we ran hindcasting simulations using model versions with different combinations of societal factors included, following the model development methodology of Wen et al.^44^. By each societal factor being either represented or not, if all data were available, we obtained 64 (2^6^) hindcasting simulations per country. For each hindcasting simulation, we quantified for different outputs (installed capacity, CO_2_ emissions and renewable generation share) the deviation between simulation outputs and historical values over the 1990–2019 period. To do so, we first calculated the SMAPE over the years, which is a relevant indicator to evaluate hindcasting performance in terms of the absolute magnitude of the deviations^29^. This indicator is also an absolute indicator without cancelation effect, which allowed us to sum the values obtained over the different technologies for installed capacity to obtain an overall accuracy indicator. We then quantified the relative performance gain or loss brought by the inclusion of societal factors in the model compared with the techno-economic model version without societal factors included. We finally identified the societal factors that contribute the most to the variability of hindcasting performance. To do so, we performed analysis of variance on hindcasting performance to compare the contributions to variance of societal factors and their interactions. We also captured the effect direction of each societal factor inclusion by comparing the average values of performance over the simulation subsets with and without the considered societal factor included^67^.

## Supplementary information

Supplementary Information Supplementary Table 1 and Figs. 1–7.

## Data Availability

Historical data about costs, generation and capacity dynamics are available via Zenodo at 10.5281/zenodo.6696776 (ref. ^68^). Other input data and sources used to represent the societal factors are described in the main text (Table 1), Methods and Supplementary Table 1. Output data from hindcasting simulations are available via Zenodo at 10.5281/zenodo.14258386 (ref. ^69^).

## Code availability

The code for the version of the STONES model used for this analysis can be obtained via Zenodo at 10.5281/zenodo.14258386 (ref. ^69^).
