# Supplementary material for: Multi-country evidence on societal factors to include in energy transition modelling
Source: Nat Energy. 2025 Feb 21;10(4):460–9. doi: 10.1038/s41560-025-01719-7 (PMC12021648; doi:10.1038/s41560-025-01719-7)
Supplement: Supplementary file 1 — Supplementary Table 1 and Figs. 1–7. [file 41560_2025_1719_MOESM1_ESM.pdf]

nature energy

Supplementary information https://doi.org/10.1038/s41560-025-01719-7

Multi-country evidence on societal factors to include in energy transition modelling

In the format provided by the authors and unedited

Contents

1 The STONES (Socio-Technical Outlook of National Energy System) model 2

1.1 The electricity demand module 2

1.2 The dispatch module 3

1.2.1 Objective function 3

1.2.2 Adequacy equation 4

1.2.3 Electricity production-related constraints 4

1.2.4 Imports/exports constraints 5

1.2.5 Storage-related constraints 5

1.3 The capacity dynamic module 7

1.3.1 Total capacity needed to order 7

1.3.2 New investments in each technology of electricity production 8

1.4 Data sources 10

2 Additional results and figures 12

2.1 Comparison of the hindcasting performance relative to the techno-economic model for installed capacity dynamics 12

2.2 Influence of societal factors on model hindcasting performance for capacity dynamics 13

2.3 Sensitivity analysis for the representation of societal factors 14

Section 1 describes the Socio-Technical Outlook of National Energy System (STONES) model built for this study. Section 2 shows additional results and figures.

1 The STONES (Socio-Technical Outlook of National Energy System) model

1.1 The electricity demand module

The electricity demand curve module allows to obtain hourly electricity demand values for the observed year y to feed the dispatch module, and for the year y+5 to feed the capacity expansion module. It uses as input the load duration curves (hourly electricity demand profiles) for the year observed (or for the closest available one if not available) taken from Jaxa-Rozen et al[1].

To reduce calculation time because of computational constraints, the data size can be reduced by time series aggregation. Among the different existing aggregation methods, we use the clustering method to obtain representative days, which has a good performance compared to others [2, 3]. However, using only electricity demand to build representative days induces the risk to decorrelate electricity demand and seasonal weather variation influencing renewable production, leading to an inconsistent dispatch for variable renewable technologies [4]. We then associate the electricity demand profile with the hourly capacity factors profiles for variable renewable energies [5–7]. We normalize the five time series to give them the same weight for time series aggregation and then used the k-Means algorithm to obtain six representative days with different weights[8]. According to the literature, using six representative days is relevant to capture the main characteristic fluctuations of electricity demand and variable generation between seasons and time of the day [9, 10]. The 144 time slices (6 days x 24 hours) we use for dispatching are at least of the same order of magnitude as in most of the energy system simulation and integrated assessment models [11, 12] .

We then modify ex-post the obtained hourly demand values. First of all, we proportionally scale the hourly demand values to equalize the annual cumulative electricity demand of the year observed and the sum of the electricity demand from the representative days weighted

by the size of the associated cluster. Secondly, to model the future hourly electricity demand values for the year y+5, we project the peak demand value following the last 5-year average growth rate of peak demand. All the hourly values are then proportionally scaled by the same factor so the maximum demand value equals the projected peak demand value. Finally, each representative day is repeated by the number of days it represents and values are sorted in descending order to obtain the projected load duration curve for the year y+5.

1.2 The dispatch module

The dispatch module determines the system-wide cost-optimal dispatch by minimizing the sum of variable costs for all technologies while satisfying hourly electricity demand over the six representative days obtained from the electricity demand module. This optimization is done using the pyomo software package in python language and the gurobi solver, satisfying different constraints detailed below.

1.2.1 Objective function

The objective function is the sum of all variable costs over the six representative days for the year y:

$$\begin{aligned} varCosts = & \sum_c \sum_i \sum_t ((vOM_c + fuel\_cost_c / fuel\_eff_c) \times p_{c,t}/1000) \times szcluster_i) \\ & + \sum_i \sum_t fuel\_cost_{imp} \times p_{imp,t}/1000 \times szcluster_i \\ & - \sum_i \sum_t exp\_profit \times p_{exp,t}/1000 \times szcluster_i \end{aligned} \quad (1)$$

where c represents the electricity production technology, i the representative day, t the time in hour for the representative day i, vOM_c the variable operational and maintenance costs for the technology c in million EUR2019/TWh, $fuel\_cost_c$ the fuel cost for the technology c in million EUR2019/TWh, $fuel\_eff_c$ the fuel efficiency, $p_{c,t}$ the hourly electricity production from the technology c at time t in GWh, $fuel\_cost_{imp}$ the cost of electricity imports in million EUR2019/MWh, $p_{imp,t}$ the electricity imported at time t in GWh, $p_{exp,t}$ the electricity exported

at time t in GWh and $szcluster_i$ the size of the cluster associated to each representative day i in days.

1.2.2 Adequacy equation

At each time $t \in [1, 24]$ of a day, the electricity demand should be met. In case of overproduction, electricity can be exported or sent to storage units.

$$demand_t = \sum_c p_{c,t} \times (1 - own\_use_c) + p_{Line,t} - stor_{ch,t} + stor_{disch,t} \quad \forall t \tag{2}$$

where $demand_t$ represents the electricity demand in GWh at time t, own_use_c the own electricity use for the technology c, $p_{Line,t}$ the net electricity imports in GWh, $stor_{ch,t}$ the storage charge flow in GWh and $stor_{disch,t}$ the storage discharge flow in GWh.

1.2.3 Electricity production-related constraints

For every dispatchable technologies dc, the electricity production should be at each time step above (below) the minimum (maximum) load factor value multiplied by the installed capacity.

$$lf\_min_{dc} \times cap_{dc} \leq p_{dc,t} \leq lf\_max_{dc} \times cap_{dc} \quad \forall\ drc, t \tag{3}$$

where lf_min_{dc} represents the minimum load factor, lf_max_{dc} the maximum load factor, $p_{dc,t}$ the electricity production at time t, cap_{dc} the installed capacity in GW.

For variable renewable technologies vrc (pv, offshore wind, onshore wind and hydro run-of river), the electricity production is equal to the hourly load factor value multiplied by the installed capacity.

$$p_{vrc,t} = lf_{vrc,t} \times cap_{vrc} \quad \forall\ vrc, t \tag{4}$$

where $p_{vrc,t}$ the electricity production at time t, lf_{vrc} represents the hourly load factor, lf_max_{dc} the maximum load factor, cap_dC the installed capacity in GW.

The variation of electricity production between two hours for each technology is limited by the ramp rate.

$$p_{c,t+1} - p_{c,t} = r_c \times cap_c \quad \forall\, c, t \tag{5}$$

$$p_{c,t} - p_{c,t+1} = r_c \times cap_c \quad \forall\, c, t \tag{6}$$

where $p_{c,t}$ represents the electricity production at time t, r_c rthe ramp-rate for the technology c in GWh per GW of installed capacity and cap_c the installed capacity in GW.

The annual production should be below the annual production potential for each technology.

$$\sum_i \sum_t (p_{c,t}/1000 \times szcluster_i) <= potential\_annual_c \tag{7}$$

where $p_{c,t}$ represents the electricity production at time t, $szcluster_i$ the size of the cluster associated to each representative day i in days, and $potential_annual_c$ the annual potential of electricity production in TWh.

1.2.4 Imports/exports constraints

$$p_{imp,t} + p_{exp,t} \leq cap_{imp} \quad \forall\, t \tag{8}$$

$$p_{imp,t} - p_{exp,t} - p_{line,t} = 0 \quad \forall\, t \tag{9}$$

where $p_{imp,t}$ represents the electricity imports at time t in GWh, $p_{exp,t}$ the electricity exports at time t in GWh, cap_{imp} the installed transmission capacity in GWh, and $p_{line,t}$ the net imports in GWh at time t.

1.2.5 Storage-related constraints

The storage discharge and charge flows at each time t are limited by the installed capacity.

$$stor_{ch,t} \leq cap_{stor} \quad \forall\, t \tag{10}$$

$$stor_{disch,t} \leq cap_{stor} \quad \forall\, t \tag{11}$$

where $stor_{ch,t}$ represents the storage charge flow at time t in GWh, $stor_{disch,t}$ the storage discharge flow at time t in GWh, cap_{stor} the installed storage capacity in GWh, and $p_{line,t}$ the net imports in GWh at time t.

To be able to capture seasonal patterns for pumped hydro storage (PHS) with a number no_{ed} of representative days, we follow Kotzur et al [3] methodology by combining modeling of inter and intra representative days storage behavior. Each hour of the year $h \in [1, 8760]$ can be represented by a time step $t \in [1, 24]$ within a day $k \in [1, 365]$, the latter being associated with a typical day $i = f(k) \in [1, no\_ed]$

The intra representative days states of charge are defined as :

$$soc\_intra_{i,t} = soc\_intra_{i,t-1} + stor_{ch,i,t-1} \times \eta - stor_{disch,i,t-1}/\eta \quad \forall\, i, \forall t \neq 1 \tag{12}$$

$$soc\_intra_{i,1} = 0 \tag{13}$$

where $soc\_intra_{i,t}$ represents the intra-representative days state of charge at time t of the representative day i in GWh, $stor_{ch,i,t}$ the storage charge flow at time t of the representative day i in GWh, $stor_{disch,i,t}$ the storage discharge flow at time t of the representative day i and η the charging/discharging efficiency.

The inter representative days states of charge are defined as :

$$\begin{split} soc\_inter_k = soc\_inter_{k-1} + soc\_intra_{i=f(k-1),24} + \\ stor_{ch,i=f(k-1),24} \times \eta - stor_{disch,i=f(k-1),24}/\eta \quad \forall\, k \neq 1 \end{split} \tag{14}$$

where soc_inter_k represents the inter-representative days state of charge in GWh at the beginning of the day k, $stor_{ch,i,t}$ the storage charging flow at time t of the representative day i

in GWh, $stor_{disch,i,g}$ the storage discharging flow at time t of the representative day i in GWh and η the charging/discharging efficiency.

The state of the charge at the beginning of the year is supposed to be identical to the state of charge at the end of the year with a cyclicity constraint:

$$soc\_inter_1 = soc\_inter_{365} + soc\_intra_{i=f(365),24} + stor_{ch,i=f(365),24} \times \eta - stor_{disch,i=f(365),24}/\eta \quad (15)$$

The state of charge is limited by the energy storage capacity at each time step:

$$0 \leq soc\_inter_k + soc\_intra_{i=f(k),t} \leq Smax; \quad \forall\, k, \forall\, t \quad (16)$$

where soc_inter_k represents the inter-representative days state of charge in GWh at the beginning of the day k, $soc\_intra_{i=f(k),g}$ represents the intra-representative days state of charge in GWh for the corresponding representative day i and $Smax$ the maximum charging capacity in GWh.

1.3 The capacity dynamic module

The capacity dynamic module determines the evolution of installed power capacity to satisfy the projected electricity demand five years later.

1.3.1 Total capacity needed to order

We estimate every year the new capacity needed to satisfy the expected peak demand five years later, i.e the maximum hourly factor from the projected load duration curve increased by a capacity margin factor. We first determine the total remaining capacity in 5 years tracking the vintage of the installed capacity to endogeneize the retirement based on the capacity $lifetime_c$ of each technology c in years. Then, we estimate the total expected required capacity following

De Boer and Van Vuuren approach [13]:

$$Total\,expected\,required\,capacity = \sum_c expected\,capacity_c \times \frac{expected\,peak\,demand \times (1 + cap\_margin)}{\sum_c (expected\,capacity_c \times cap\_credit_c \times (1 - own\_use_c)} \quad (17)$$

The total capacity to order to satisfy the expected peak demand is then equal to the difference between the total expected required capacity and the total expected remaining capacity.

1.3.2 New investments in each technology of electricity production

First the expected hourly production of variable renewable technologies (PV, hydro run-of river, onshore & offshore wind) is subtracted from the load duration curve to obtain the hourly electricity demand over the year net of expected variable renewable contribution. Then, because only a part of the installed capacity generates electricity at full load all year, we follow previous approaches [13–15] by dividing the load duration curve into investment segments (i.e peak load, intermediate load, base load...) to make the link with dispatch decisions. For each investment segment, technologies are compared based on their annualized levelized costs of energy (equation 18). The total capacity to order is split between technologies with a multinomial logit equation (equation 19) which is a common approach in energy simulation models [13, 16]. Finally, the capital stock evolves at each time step for each technology based on new investments made and retirement of the existing capacity according to the lifespan.

$$LCOE_{l,c} = \frac{inv\_cost_c \times \frac{d}{1-(1+d)^{-lspan}} + fixed\_cost}{24 \times 365 \times lf_c \times df_l} + vOM_c + fuel\_cost_c/fuel\_eff_c \quad (18)$$

where inv_costs_c represents the investments costs in EUR2019/kW, $fixed\_cost_c$ the fixed annual cost in EUR2019/kW, vOM_c the variable operational and maintenance costs in EUR2019/kWh, $fuel\_cost_c$ the fuel cost in EUR2019/kWh, $fuel\_eff_c$ the fuel efficiency, d the discount rate, $lspan$ the lifespan of the installed capacity, lf_c the load factor for the technology

c and df_l the annual power generation ratio for the load segment of investment l.

$$order\_cap_{l,c} = tot\_order\_cap \times \frac{size\_seg_l}{\sum_{l=0}^{n} size\_seg_l} \times \frac{LCOE_{l,c}^{-\lambda}}{\sum_c LCOE_{l,c}^{-\lambda}} \tag{19}$$

where $order\_cap_{l,c}$ represents the new investments for the technology c in the the load segment of investments l in GW, tot_order_cap the total capacity to order to satisfy the expected peak demand five years later in GW, $size\_seg_l$ the size of the investment load segment l in GW, $LCOE_{l,c}$ the levelized cost of energy for the technology c and the investment load segment l and λ the cost sensitivity of investment choices.

1.4 Data sources

Supplementary Table 1: Data sources used to complement Jaxa-Rozen et al. [1]

**Variable**	**Source**
Coal capacity vintage	Global Energy Monitor [17]
Gas capacity vintage	Global Energy Monitor [18]
Nuclear capacity vintage	Kanellopoulos et al. [19]
Technical potential of pumped hydro storage installed capacity	Geth et al. [20]
Public opinion on nuclear power	*Public opinion in the European Community on Energy in 1991; Eurobarometer 39.1, European Opinion and Energy Matters 1993; Eurobarometer 46, European Opinion and Energy Matters 1997; Eurobarometer 58.0, Europeans and Biotechnology in 2002; Special Eurobarometer 262; Flash Eurobarometer 206a; Special Eurobarometer 341*; European Commission [21];<br>Kim et al. [22]
Public opinion on wind, solar, coal- and gas-based generation	*Special Eurobarometer 262; Special Eurobarometer 364*; European Commission [21]<br>European Social Survey European Research Infrastructure [23]
Weighted average costs of capital for power technologies	Polzin et al. [24] and correspondence with authors
Governance indicators: entry barriers and public ownership	OECD Product Market Regulation database [25]

2 Additional results and figures

2.1 Comparison of the hindcasting performance relative to the techno-economic model for installed capacity dynamics

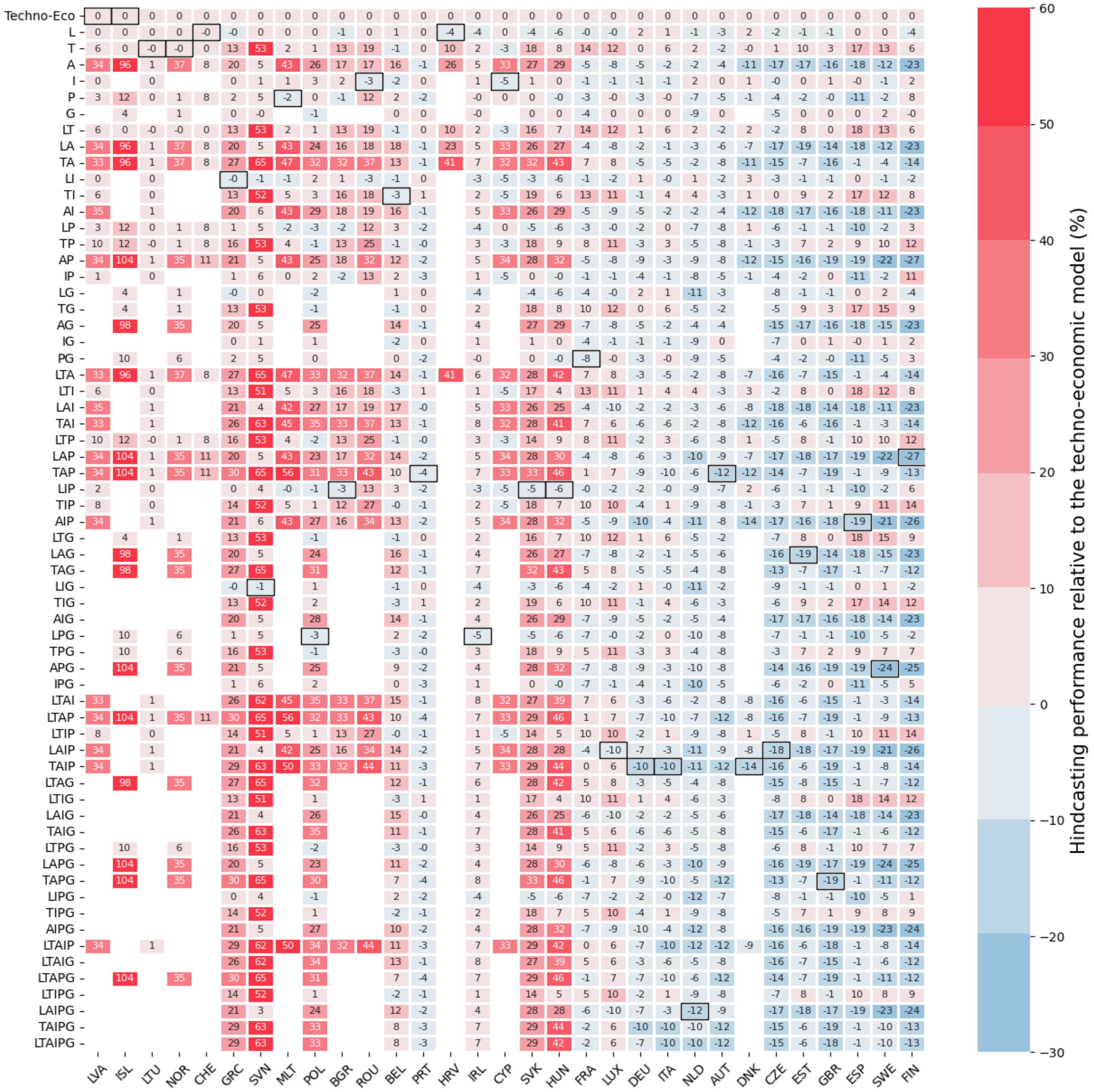

Supplementary Figure 1: Comparison of the hindcasting performance relative to the techno-economic model for installed capacity according to the societal factors included. Hindcasting performance corresponds to the symmetric mean absolute percentage error (SMAPE) summed over all technologies. A negative value corresponds to a lower deviation from historical observations than the techno-economic model, meaning a better performance than the techno-economic model. Each letter refers to one societal factor incorporated: L — Lock-in; T — Fast Transition; A — Actors heterogeneity; I — Investment risk; P — Public acceptance; G — Governance. Empty cells correspond to cases where country data were not available for one of the societal factor of the combination. The best performance for each country is highlighted by a black frame.

2.2 Influence of societal factors on model hindcasting performance for capacity dynamics

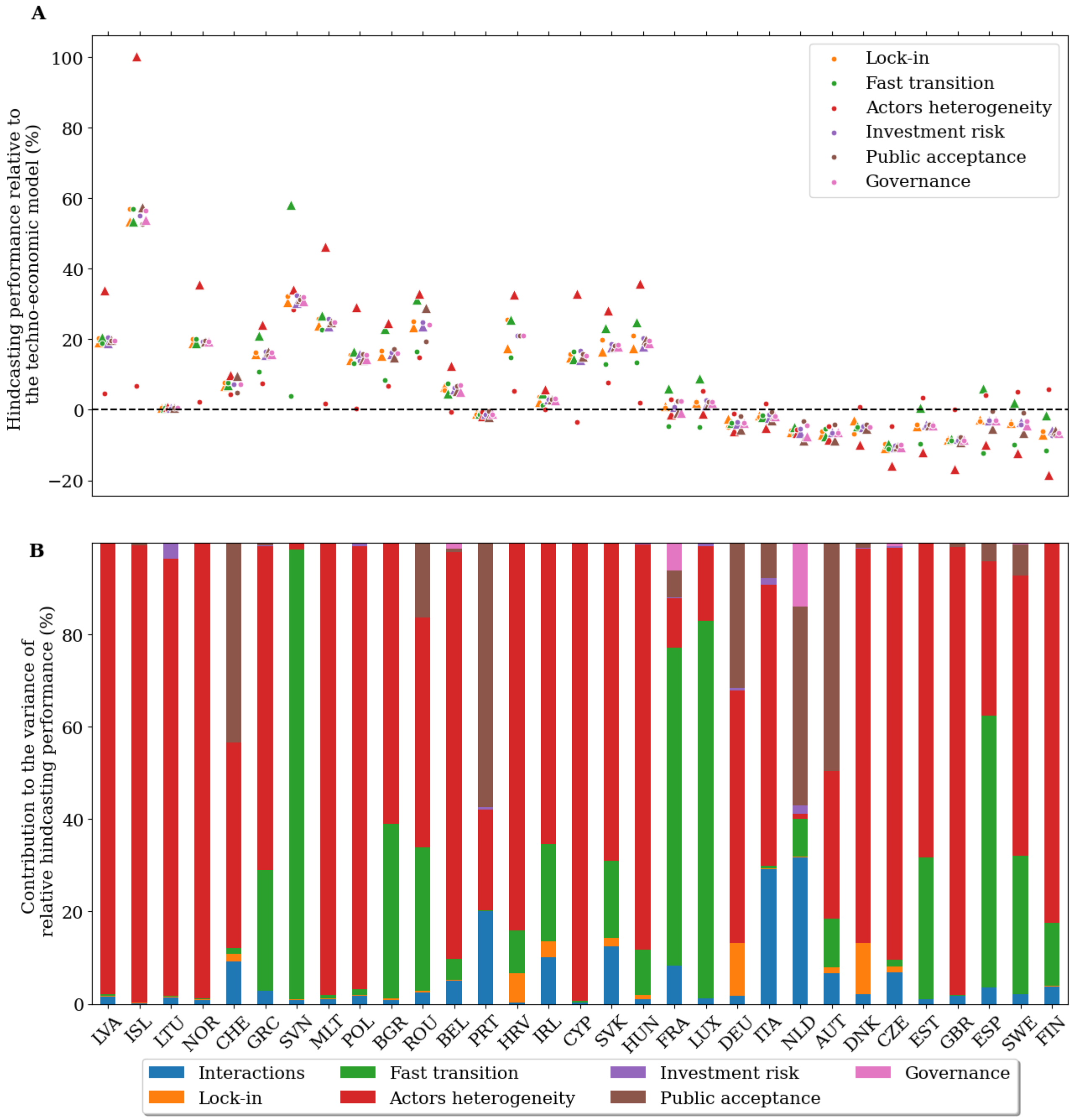

Supplementary Figure 2: Influence of societal factors on model hindcasting performance for capacity dynamics. Upper figure (a) shows the direction of the effect of each societal factor. Bottom figure (b) shows the analysis of variance (ANOVA) on model hindcasting performance. Hindcasting performance corresponds to the symmetric mean absolute percentage error (SMAPE) summed over all technologies. In figure (a), triangle represents the average relative model performance across the hindcasting simulation subset with the considered societal factor included. Point represents the average relative model performance across the hindcasting simulation subset without the considered societal factor included.

2.3 Sensitivity analysis for the representation of societal factors

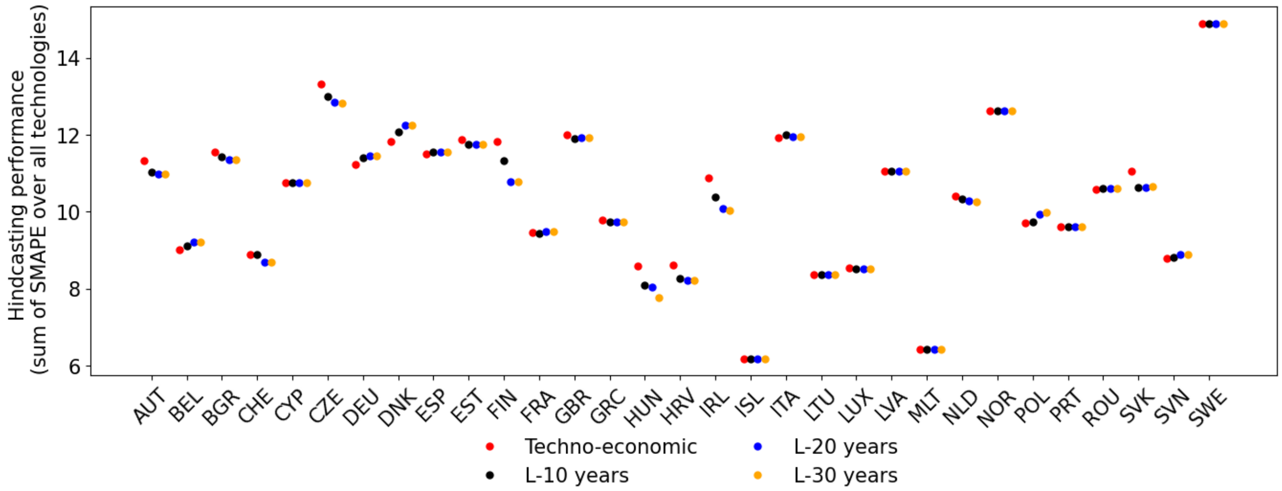

Supplementary Figure 3: Sensitivity analysis for the *Lock-in* societal factor. Variations are made on the maximum lifespan extension of power capacity. The black point represents the version of the societal factor included in the main analysis.

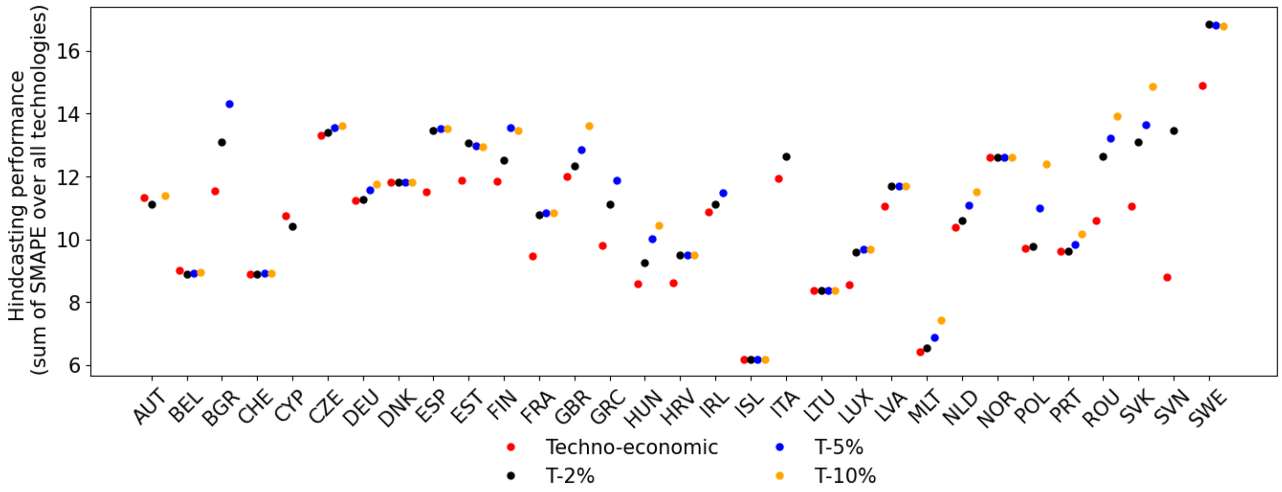

Supplementary Figure 4: Sensitivity analysis for the *Fast transition* societal factor. Variations are made on the annual limit of early retired capacity, for each technology, in percentage of the total installed capacity. The black point represents the version of the societal factor included in the main analysis. Missing points correspond to cases where the model is unable to produce a scenario until the end of the time period.

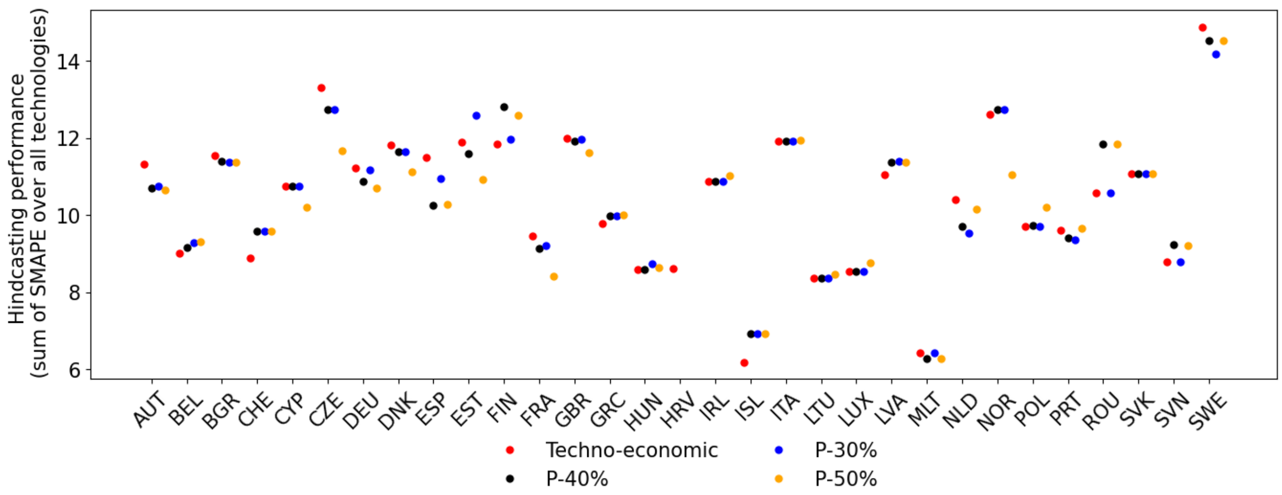

Supplementary Figure 5: Sensitivity analysis for the *Public acceptance* societal factor. Variations are made on the population threshold supporting a technology, below which the technology in question can no longer be built. The black point represents the version of the societal factor included in the main analysis. Missing points correspond to countries for which acceptance data were not available.

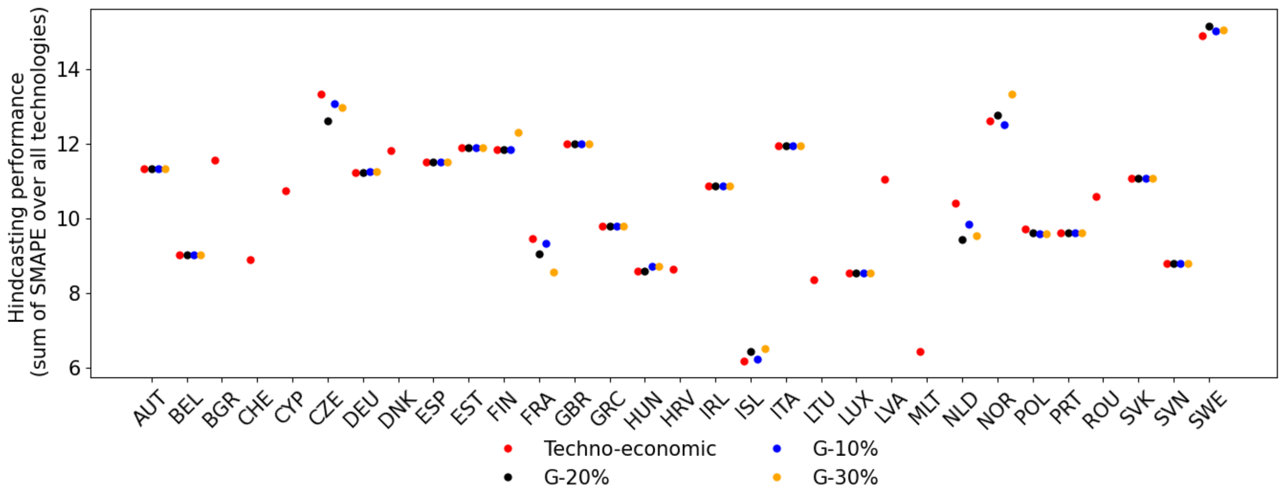

Supplementary Figure 6: Sensitivity analysis for the *Governance* societal factor. Variations are made on the relative reduction (increase) of renewable investment cost when entry barriers are low (high) and public ownership is high (low). The black point represents the version of the societal factor included in the main analysis. Missing points correspond to countries for which governance data were not available.

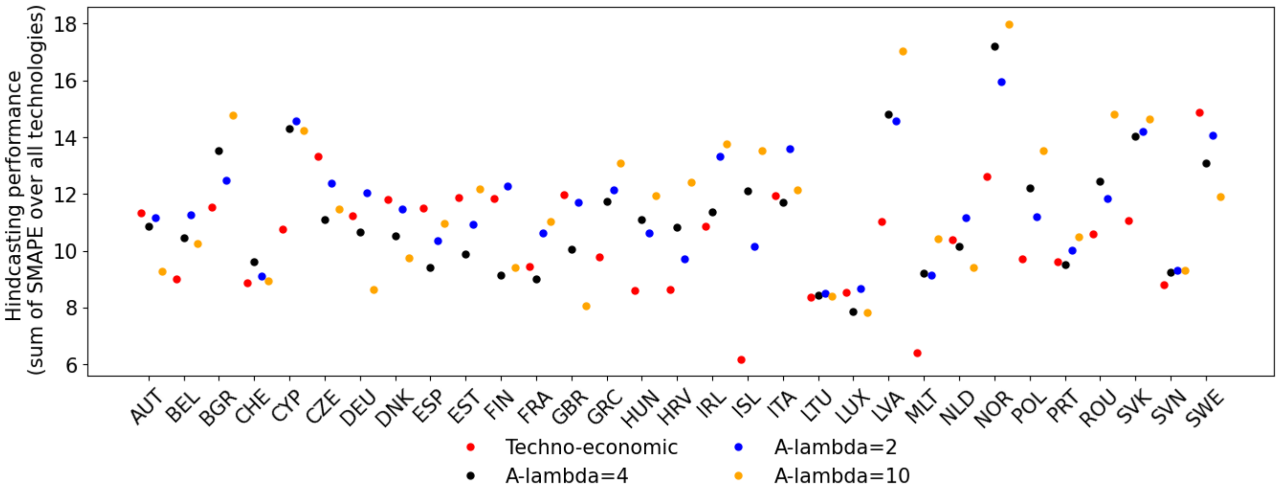

Supplementary Figure 7: Sensitivity analysis for the *Actors heterogenety* societal factor. Variations are made on the lambda parameter of the multinomial logit equation for investment choices to modify the cost sensitivity. A lambda value of 4 corresponds to a case where, between two technologies, a cost saving of 20% results in a 75%. A lambda value of 2 corresponds to a case where a cost saving of 20% results in a 60% market share. A lambda value of 10 corresponds to a case where a cost saving of 20% results in a 90% market share. The black point represents the version of the societal factor included in the main analysis.

References

1. Jaxa-Rozen, M., Wen, X. & Trutnevyte, E. Historic data of the national electricity system transitions in Europe in 1990–2019 for retrospective evaluation of models. en. *Data in Brief* **43,** 108459. ISSN: 2352-3409. https://www.sciencedirect.com/science/article/pii/S2352340922006540 (2023) (Aug. 2022).

2. Marcy, C., Goforth, T., Nock, D. & Brown, M. Comparison of temporal resolution selection approaches in energy systems models. en. *Energy* **251,** 123969. ISSN: 0360-5442. https://www.sciencedirect.com/science/article/pii/S0360544222008726 (2023) (July 2022).

3. Kotzur, L., Markewitz, P., Robinius, M. & Stolten, D. Time series aggregation for energy system design: Modeling seasonal storage. en. *Applied Energy* **213,** 123–135. ISSN: 0306-2619. https://www.sciencedirect.com/science/article/pii/S0306261918300242 (2022) (Mar. 2018).

4. Pfenninger, S. Dealing with multiple decades of hourly wind and PV time series in energy models: A comparison of methods to reduce time resolution and the planning implications of inter-annual variability. en. *Applied Energy* **197,** 1–13. ISSN: 0306-2619. https://www.sciencedirect.com/science/article/pii/S0306261917302775 (2022) (July 2017).

5. De Felice, M. *ENTSO-E Pan-European Climatic Database (PECD 2021.3) in Parquet format* Type: dataset. Oct. 2022. https://zenodo.org/record/7224854 (2023).

6. Pfenninger, S. & Staffell, I. Long-term patterns of European PV output using 30 years of validated hourly reanalysis and satellite data. en. *Energy* **114,** 1251–1265. ISSN: 0360-5442. https://www.sciencedirect.com/science/article/pii/S0360544216311744 (2023) (Nov. 2016).

7. Staffell, I. & Pfenninger, S. Using bias-corrected reanalysis to simulate current and future wind power output. en. *Energy* **114,** 1224–1239. ISSN: 0360-5442. https://www.sciencedirect.com/science/article/pii/S0360544216311811 (2023) (Nov. 2016).

8. Kittel, M., Hobbie, H. & Dierstein, C. Temporal aggregation of time series to identify typical hourly electricity system states: A systematic assessment of relevant cluster algorithms. en. *Energy* **247,** 123458. ISSN: 0360-5442. https://www.sciencedirect.com/science/article/pii/S0360544222003619 (2023) (May 2022).
9. Després, J. *et al.* POLES-JRC model documentation. *JRC Europa.* https://publications.jrc.ec.europa.eu/repository/bitstream/JRC113757/kjna29454enn.pdf (2024) (2018).
10. Nahmmacher, P., Schmid, E., Hirth, L. & Knopf, B. Carpe diem: A novel approach to select representative days for long-term power system modeling. en. *Energy* **112,** 430–442. ISSN: 0360-5442. https://www.sciencedirect.com/science/article/pii/S0360544216308556 (2022) (Oct. 2016).
11. Pietzcker, R. C. *et al.* System integration of wind and solar power in integrated assessment models: A cross-model evaluation of new approaches. en. *Energy Economics* **64,** 583–599. ISSN: 0140-9883. https://www.sciencedirect.com/science/article/pii/S0140988316303395 (2021) (May 2017).
12. Cole, W. *et al. Variable renewable energy in long-term planning models: a multi-model perspective* tech. rep. (National Renewable Energy Lab.(NREL), Golden, CO (United States), 2017). https://www.osti.gov/biblio/1416124 (2024).
13. De Boer, H. S. (S.) & van Vuuren, D. (P.) Representation of variable renewable energy sources in TIMER, an aggregated energy system simulation model. en. *Energy Economics* **64,** 600–611. ISSN: 0140-9883. https://www.sciencedirect.com/science/article/pii/S0140988316303528 (2023) (May 2017).
14. Wise, M. *et al.* Representing power sector detail and flexibility in a multi-sector model. en. *Energy Strategy Reviews* **26,** 100411. ISSN: 2211-467X. https://www.sciencedirect.com/science/article/pii/S2211467X1930104X (2022) (Nov. 2019).
15. Després, J. *et al.* Storage as a flexibility option in power systems with high shares of variable renewable energy sources: a POLES-based analysis. en. *Energy Economics* **64,**

638–650. ISSN: 0140-9883. https://www.sciencedirect.com/science/article/pii/S0140988316300445 (2021) (May 2017).

16. Keppo, I. *et al.* Exploring the possibility space: taking stock of the diverse capabilities and gaps in integrated assessment models. en. **16.** Publisher: IOP Publishing, 053006. ISSN: 1748-9326. https://doi.org/10.1088/1748-9326/abe5d8 (2021) (Apr. 2021).

17. Global Energy Monitor. *Global Coal Plant Tracker* 2021. https://globalenergymonitor.org/projects/global-coal-plant-tracker.

18. Global Energy Monitor. *Global Gas Plant Tracker* 2021. https://globalenergymonitor.org/projects/global-gas-plant-tracker/.

19. Kanellopoulos, K., De Felice, M., Hidalgo Gonzalez, I. & Bocin, A. *JRC Open Power Plants Database (JRC-PPDB-OPEN)* Version Number: 1.00 Type: dataset. Dec. 2019. https://zenodo.org/record/3574566 (2023).

20. Geth, F., Brijs, T., Kathan, J., Driesen, J. & Belmans, R. An overview of large-scale stationary electricity storage plants in Europe: Current status and new developments. en. *Renewable and Sustainable Energy Reviews* **52,** 1212–1227. ISSN: 1364-0321. https://www.sciencedirect.com/science/article/pii/S1364032115007923 (2023) (Dec. 2015).

21. European Commission. *Eurobarometer* 2022. https://europa.eu/eurobarometer/screen/home.

22. Kim, Y., Kim, M. & Kim, W. Effect of the Fukushima nuclear disaster on global public acceptance of nuclear energy. en. *Energy Policy* **61,** 822–828. ISSN: 0301-4215. https://www.sciencedirect.com/science/article/pii/S0301421513006149 (2023) (Oct. 2013).

23. European Social Survey, E. S. S. E. R. I. *ESS8 - integrated file, edition 2.2 [Data set]* 2020. https://doi.org/10.21338/ESS8E02_2.

24. Polzin, F. *et al.* The effect of differentiating costs of capital by country and technology on the European energy transition. en. *Climatic Change* **167,** 26. ISSN: 1573-1480. https://doi.org/10.1007/s10584-021-03163-4 (2021) (July 2021).

25. OECD. Product Market Regulation Network Sectors indicators: Methodology for calculating the 1975-2018 time series. en (2018).
